# Workforce, succession planning, and optimism for the future among Swiss general practitioners with additional training in complementary medicine in 2016: A nationwide cross-sectional study

**DOI:** 10.1371/journal.pone.0342226

**Published:** 2026-02-06

**Authors:** Erschena Reichle, Martin Frei-Erb, Sven Streit

**Affiliations:** 1 Institute of Complementary and Integrative Medicine (IKIM), University of Bern, Bern, Switzerland; 2 Institute of Primary Health Care (BIHAM), University of Bern, Bern, Switzerland; Endeavour College of Natural Health, AUSTRALIA

## Abstract

The shortage of general practitioners (GPs) and the aging workforce of established GPs in Switzerland are persistent. Furthermore, data on GPs with additional training in complementary medicine (CM) are lacking. This study aimed to obtain information on GPs with training in CM. A nationwide cross-sectional study was conducted using an anonymous online survey to assess the demographics, practice structures, workloads, and future perspectives. The participants were members of four CM societies representing doctors with CM certificates (n = 1,067) in traditional Chinese medicine/acupuncture (TCM/A), homeopathy (HOM), anthroposophical medicine, and phytotherapy. Overall, 206 GPs were included and divided into three groups: TCM/A (n = 73), HOM (n = 76), and others (n = 57). Most participants were male, and approximately half worked in double or group practices. They worked an average of 36 h per week, which had decreased by 13 h over the past 5 years, with plans for further reduction in the future. Approximately 18% planned to continue working beyond the retirement age, and 4% wanted to retire before that age. The majority expressed optimism about the future of CM in Swiss healthcare but less for individual CM disciplines. Compared to the 2015 Swiss Workforce Study, more CM GPs were female, worked less often in group practices, made fewer house calls, and were more optimistic about the future. CM-GPs in Switzerland face the same challenges as conventional GPs. National action and collaboration are needed to improve working conditions and to address the trend towards part-time work and an ageing workforce to safeguard healthcare for the population.

## Introduction

The shortage of general practitioners (GPs) and associated aging of established GPs have been an issue in many European countries, including Switzerland [1]. The Swiss Workforce Study found that the mean age of GPs was increased from 46 years in 1993–55 years in 2015 [2]. Almost half of the participants indicated their intention to work beyond the traditional retirement age of 65 years, and 15% of the current workforce comprised doctors who were over 65 years old. The foreseeable retirement of these doctors in 5–10 years will exacerbate the shortage of GPs and offer the opportunity for structural change towards more group practices. In 2015, only 26% of Swiss GPs were female, 94% were self-employed, and 32% worked in group practices with more than 2 doctors [2]. Young GPs would prefer to work part-time in small GP-owned group practices with up to six doctors, initially as employees and later as associates [3]. Figures from the Canton of Bern indicate a trend in this direction, with a 10% increase (from 46 to 56%) in doctors working in group practices between 2013 and 2017 [4].

Swiss GPs can acquire additional diagnostic and therapeutic skills during or after their postgraduate training. Since 1999, complementary medicine (CM) has been incorporated into these structured training courses, which are approved by the Swiss Institute of Medical Education and include anthroposophical medicine, homeopathy, phytotherapy, and traditional Chinese medicine/acupuncture (TCM/A) [5].

After a broad political discussion, including a health technology assessment (2001–2005) and a nationwide referendum in 2009, in which 67% of voters were in favor of more support from CM, services for CM doctors with proficiency certificates have been reimbursed by mandatory basic insurance since 2017 [6,7]. As part of the health technology assessment, a national survey on the workforce and practice structure was conducted in 2001 among GPs with and without CM [6]. CM doctors were more often female, worked part-time, and practiced in group settings.

Data equivalent to those from the Swiss Workforce Study are lacking for doctors with additional training in CM. They likely face similar challenges as conventional doctors, given their primary care roles [6]. To obtain more information, we conducted a survey with the following objectives: 1) analyze the number of Swiss doctors who have received additional training in CM (anthroposophical medicine, homeopathy, phytotherapy, and TCM/A) and who practice in primary care, including the structure of the workplace/clinical activity and possible future changes, current workload, and potential changes in the coming years; 2) investigate plans/problems in recruiting younger staff as successors; and 3) to evaluate the optimism about the future of CM in the Swiss healthcare system.

## Materials and methods

As part of a master’s thesis protocol, a questionnaire similarly to the 2015 Swiss Workforce Study was developed to conduct a nationwide cross-sectional study using an anonymous online survey from November 17, 2017, to April 30, 2018 [8] and S1 Questionnaire]. The study was conducted as part of a doctoral dissertation at the University of Bern.

### Study population

All doctors (GPs, specialists) with a proficiency certificate in complementary medicine (TCM/A, homeopathy, phytotherapy, anthroposophical medicine) practicing in Switzerland could participate in this study. However, we have limited our analysis to GPs due to the presence of a comparison group (Swiss Workforce Study) [2]. For data protection reasons, we were unable to obtain the addresses of all doctors from the Swiss Medical Association. Therefore, all members of the four medical societies (the VAOAS, SVHA, SMGP, and ASA) were invited to participate [9–12]. In the absence of compulsory membership, they do not represent all doctors with additional training in CM, but they do represent the majority. A comparison of the number of doctors with proficiency certificates and the number of members of the corresponding society in 2017 showed the following figures: TCM/A 598 vs 682, HOM 204 vs 291, PT/AM 155 vs 140 [13].

### Questionnaire

The questionnaire was structured similarly to the 2015 Swiss Workforce Study, allowing for a comparison of the results [S1 Questionnaire]. The first part included the doctors’ demographic data, including age and sex, as well as information on practice structure, workload, and retirement planning. The question about the reduction of working hours in the last 5 years (question 51/52) referred to changes in planned weekly working time, operationalised as reduced attendance in the doctor’s office, and not to the exact number of working hours. In Swiss primary care, reductions in workload are typically implemented in discrete units (e.g., half or full working days). A reduction of half a working day corresponds to approximately 4 hours less presence per week.

We expanded the survey to include questions regarding the recruitment of young professionals and optimism regarding the development of complementary medicine in the Swiss healthcare system. An online survey was created and conducted using Unipark® by two authors (ER and MFE). Unipark® is a web-based survey software developed by Tivian® specifically designed for students and academic institutions [14]. Improvements were made by doctors and scientific staff of the Institute for Complementary and Integrative Medicine. The questionnaire was translated into French by native speakers. Translation to Italian was waived because it is reasonable to assume that Swiss academics generally have proficiency in a second national language. Finalization took place after a second test run with an online link in both languages.

### Procedure

In November 2017, we sent an invitation letter with a link to the online survey to all 1,113 members (GPs and specialists) practicing in Switzerland, in either German or French, to match their language of residence. For doctors who refused or were unable to complete the questionnaire online, it was enclosed in a paper form with a stamped reply envelope. To motivate participation, a circular email was sent by the presidents of the professional societies to their members, followed by a reminder letter at the end of December 2017 and March 2018. The survey was closed on April 30, 2018.

### Ethics

Under Swiss ethics guidelines, the study did not require formal ethics approval due to the anonymity of the participants [15].

### Informed consent statement

Participation in the study was voluntary. According to Swiss regulations for research involving anonymized survey data without sensitive personal information, explicit written or verbal consent is not required. Completion and return of the questionnaire were considered to constitute informed consent to participate, in line with Swiss human research legislation (Humanforschungsgesetz, HFG Art. 2, Abs. 2).

### Data analysis

The Statistical Package for Social Sciences (IBM SPSS, version 28.0) was used to analyze the data [16]. The study population was categorized into three groups: TCM/A, HOM, and Others. The “Others” category comprised participants with qualifications in phytotherapy and anthroposophical medicine and those with multiple qualifications in CM. This grouping was necessary because the number of participants in these subcategories was insufficient for individual analyses. Descriptive statistics were used to summarize the responses and present the results of the general analysis and subgroups as percentages or means and standard deviations, where appropriate. A chi-square test was conducted to analyze categorical data concerning the characteristics of the study group, the practice structure, workload, professional commitment, and succession planning. Linear regression was used for continuous data, including age, years, the number of doctors, days per year, and hours per week.

Owing to the predominantly quantitative nature of the 2015 Swiss Workforce Study data, which are presented as percentages and median values, a comparison between these data points was feasible. Working hours, quantified in hours in this survey and as half-days in the 2015 Swiss Workforce Study, were adjusted by defining 4 h as equivalent to 1 half-day. To facilitate a meaningful comparison with the data on optimism for the future, the four-stage graduation model (very, rather, little, not at all) was divided into two stages (very/rather, little/not at all).

Specific data were exported from SPSS into Excel.xlsx to generate Figs 2–7.

**Fig 1:**
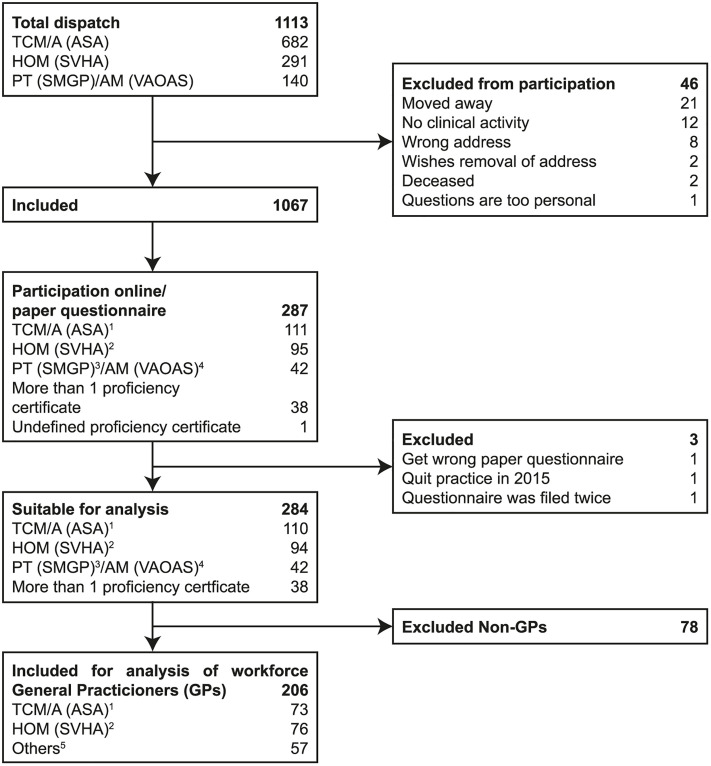
Study Flowchart, starting from the doctors contacted to the doctors included in the analysis. ^1^Traditional Chinese Medicine/Acupuncture. ^2^Homeopathy. ^3^Phytotherapy. ^4^Anthroposophical Medicine. ^5^AM, PT, holding > 1 proficiency certificate.

**Fig 2:**
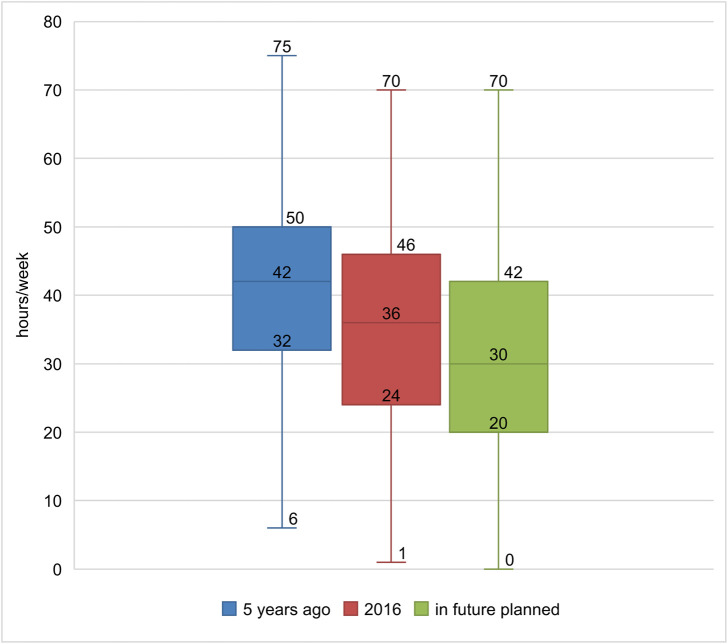
Boxplot: Development of working hours over time of the whole study sample (n = 206).

**Fig 3:**
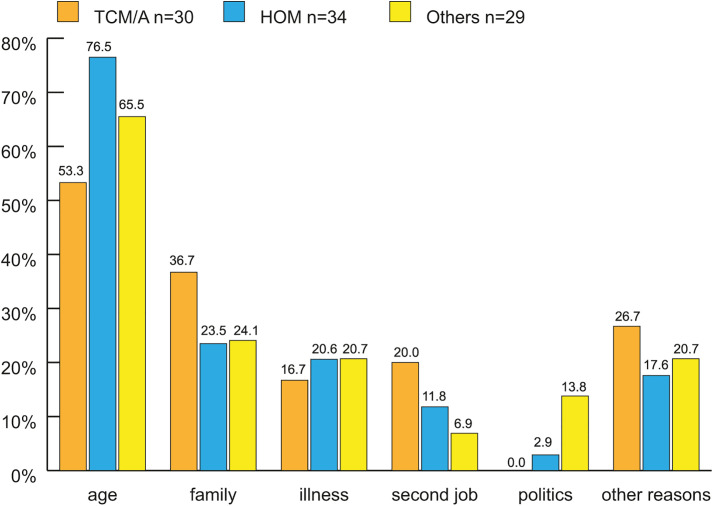
Reasons why working hours have been reduced, in subgroups (n = 93^1,2^). ^1^93 of 206 Participants (45.1%) have reduced their working hours and have been ask for reasons. The reasons are mentioned above (x axis), multiple selection was possible. ^2^100% refers to the subgroups, since multiple selection was possible the sum exceeds 100% in each subgroup (n = 142 answers). TCM/A: Traditional Chinese Medicine/Acupuncture. HOM: Homeopathy. Others: Anthroposophical Medicine, Phytotherapy, GPs holding > 1 proficiency certificate.

**Fig 4:**
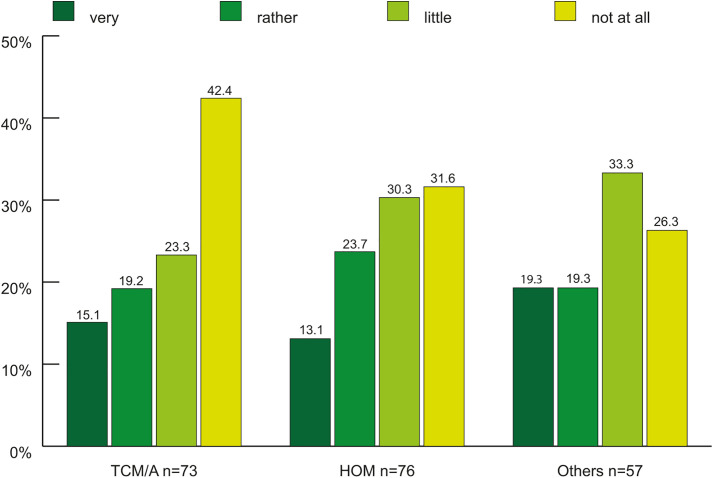
Worrying about succession across subgroups in percent (n = 206^1,2^). ^1^each subgroup was analyzed (TCM/A = 73, HOM = 76, Others = 57). In the subgroups “Others” and HOM each 1 case was NA (Non-Applicable) but was included in the count. ^2^100% refers to each subgroup. In “Others” 1 case was NA and explains the missing 1.8%. In HOM 1 case was NA and explains the missing 1.3%. TCM/A: Traditional Chinese Medicine/Acupuncture. HOM: Homeopathy. Others: Anthroposophical Medicine, Phytotherapy, GPs holding > 1 proficiency certificate.

**Fig 5:**
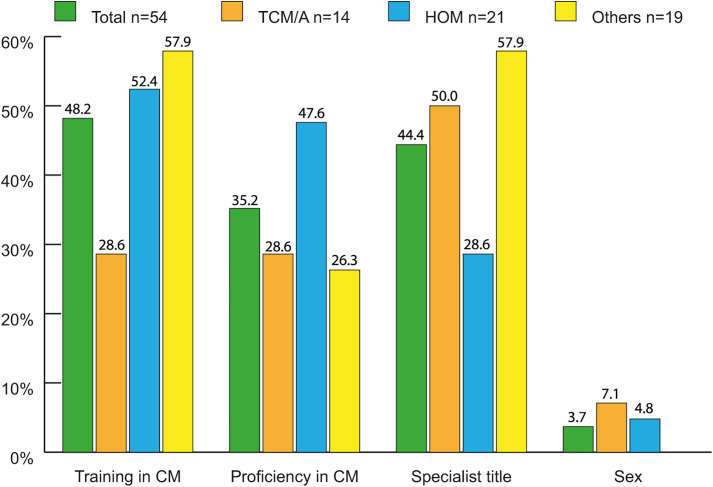
Importance of criteria during the search for succession from participants looked/looking for successor(s) in percent (n = 54^1,2^). ^1^54 have looked for a successor(s) and therefore have been considered for this question. In the survey participants were listed 6 criteria to scale (not/less/quite important/important) in question Nr. 64, criteria participants have selected as important were evaluated in this graph. 2 criterias (age, nationality) have not been selected at all. ^2^100% refers to each subgroup. TCM/A: Traditional Chinese Medicine/Acupuncture. HOM: Homeopathy. Others: Anthroposophical Medicine, Phytotherapy, GPs holding > 1 proficiency certificate. CM: Complementary Medicine.

**Fig 6:**
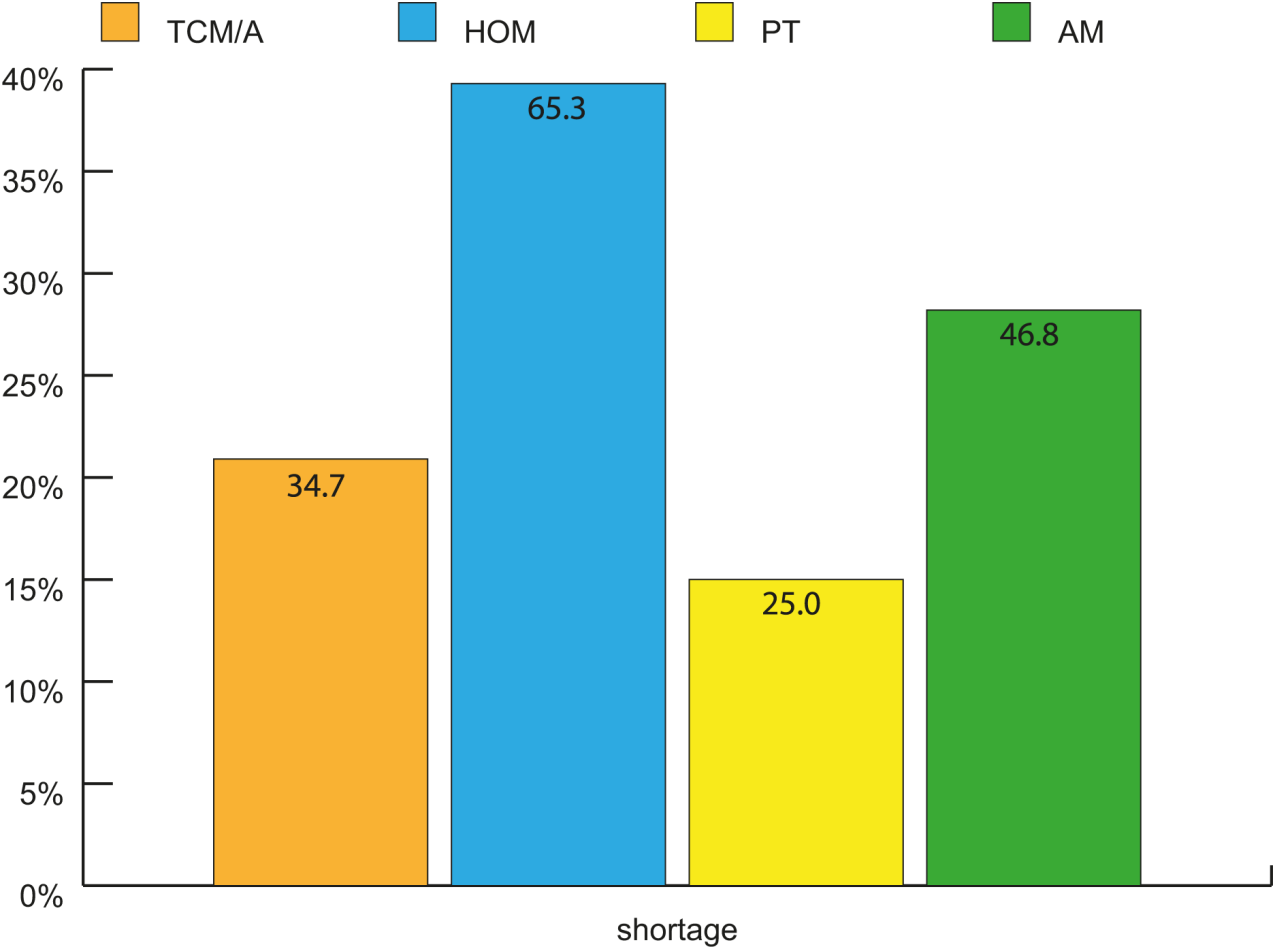
Shortage of CM-Disciplines in regional healthcare, estimated from participants confirming to have a shortage in CM in their region in percent (n = 124^1,2^). ^1^124 participants confirmed to have a shortage of physicians in CM in their region and have specified the shortage in 4 CM specialties. Multiple selection was possible. ^2^100% refers to n = 124 for each bar, since multiple selection was possible the sum exceeds 100%. TCM/A: Traditional Chinese Medicine/Acupuncture. HOM: Homeopathy. PT: Phytotherapy, GPs holding > 1 proficiency certificate. AM: Anthroposophical Medicine.

**Fig 7:**
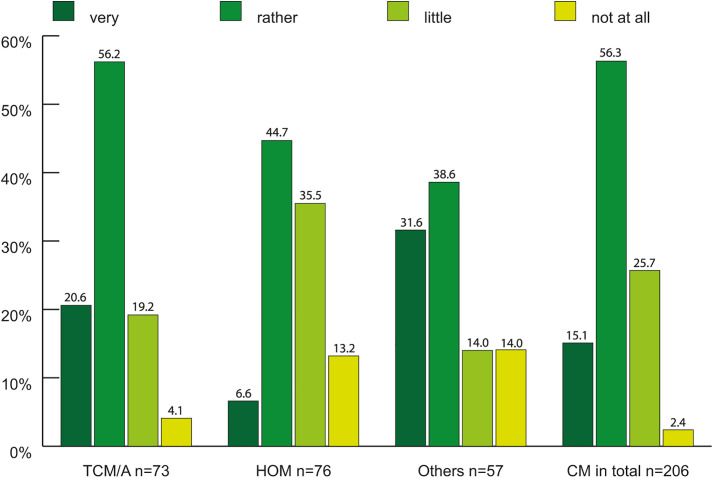
Optimism about the future of individual disciplines and CM as a whole, estimated from each subgroup for their discipline and from the whole study sample for CM in general in percent (n = 206^1,2^). ^1^each subgroup was analyzed for their specific CM-discipline (TCM/A = 73, HOM = 76, Others = 57), CM as a whole was analyzed using the whole study sample (n = 206). In “Others” 1 case was NA (Non-Applicable) but was included in the count. ^2^100% refers to each subgroup/the whole study sample. In “Others” 1 case was NA and explains the missing 1.8%. TCM/A: Traditional Chinese Medicine/Acupuncture. HOM: Homeopathy. Others: Anthroposophical Medicine, Phytotherapy, GPs holding > 1 proficiency certificate. CM: Complementary Medicine.

## Results

A total of 1,067 of the 1,113 physicians who were contacted were included in the study population for the survey (Fig 1). Of these, 284 (270 online, 14 paper questionnaires) were eligible for analysis (27%). After excluding non-GPs, 206 individuals (19%) were categorized into three subgroups according to their qualifications in CM: TCM/A (n = 73, 35%), HOM (n = 76, 37%), and Others (n = 57, 28%).

### Characteristics

The comparison of the three groups reveals significant disparities in age (p = 0.025), employment status (p = 0.008), and the focus of their practice (p < 0.001) (Table 1). HOM GPs are older and mainly work self-employed. Almost half of TCM/A GPs practice predominantly CM and not the combination of CM and CON like the other two groups. The significant difference in the time since graduation in CM is due to the different timing of the introduction of certificates (1999 vs 2011 for phytotherapy).

**Table 1:** Characteristics of general practitioners in the study sample (n = 206).

Characteristics	Total n = 206	TCM/A^1^ n = 73 (35.4%)	HOM^2^ n = 76 (36.9%)	Others^3^ n = 57 (27.7%)	p-value*
Age, years, mean (SD)	59.1 (8.7)	55.9 (9.8)	62.5 (5.7)	58.8 (8.9)	0.025^b^
Gender, no. (% per column)					
Female	79 (38.3%)	32 (43.8%)	30 (39.5%)	17 (29.8%)	0.256^a^
Male	127 (61.7%)	41 (56.2%)	46 (60.5%)	40 (70.2%)	
Nationality, no. (% per column)					
Switzerland	173 (84%)	65 (89%)	63 (82.9%)	45 (78.9%)	0.614^a^
Germany	24 (11.7%)	6 (8.2%)	9 (11.8%)	9 (15.8%)	
Other countries^4^	9 (4.3%)	2 (2.8%)	4 (5.3%)	3 (5.3%)	
Years since graduation CM^5^, mean (SD) 1 Case is NA	17.4 (7.1) (n = 205)	18.8 (4.7)	18.6 (6.9)	13.5 (8.4) (n = 56)	<0.049^b^
Employment status, no. (% in column)					
Self-employed	185 (89.8%)	62 (84.9%)	74 (97.4%)	49 (86%)	0.008^a^
Employed	20 (9.7%)	11 (15.1%)	1 (1.3%)	8 (14%)	
NA	1 (0.5%)	–	1 (1.3%)	–	
Region, no. (% per column)					
Rural region < 5’000	37 (18.0%)	13 (17.8%)	17 (22.4%)	7 (12.3%)	0.487^a^
Small town 5’000–10’000	48 (23.3%)	16 (21.9%)	15 (19.7%)	17 (29.8%)	
Urban region > 10’000	120 (58.2%)	44 (60.3%)	43 (56.6%)	33 (57.9%)	
NA	1 (0.5%)	–	1 (1.3%)	–	
Focus, no. (% in column)					
Mainly CM	58 (28.2%)	31 (42.5%)	20 (26.3%)	7 (12.3%)	<0.001^a^
CON^6^ and CM	135 (65.5%)	34 (46.6%)	55 (72.4%)	46 (80.7%)	
Mainly CON^6^	12 (5.8%)	8 (10.9)	1 (1.3%)	3 (5.3%)	
NA	1 (0.5%)	–	–	1 (1.7%)	

*Statistical analysis is conducted using SPSS; NA (Non-Applicable) data points, if present, are always included in calculations.

^a^Chi-Square Test (Pearson Chi-Square, 2-sided).

^b^Linear Regression ANOVA.

^1^Traditional Chinese Medicine/Acupuncture.

^2^Homeopathy.

^3^Anthroposophical Medicine, Phytotherapy, holding > 1 proficiency certificate.

^4^France/Italy/Austria/others.

^5^CM Complementary Medicine.

^6^CON Conventional Medicine.

### Practice structure

The comparison of the practice structure shows few differences (Table 2). Almost half of the CM GPs work in a dual or group practice with a significant difference between the 3 groups (p < 0.001). The majority of HOM GPs work in a single practice. HOM GPs and participants in the Others subgroup were significantly more likely to work with other CM doctors in a double or group practice than TCM/A GPs (p = 0.001). On average, doctors had been working in the same practice for 18 years; however, approximately 40% had changed their practice type within the last 5 years, mostly from a double or group practice to a single practice. In terms of child and adolescent care, TCM/A GPs offered significantly less care (p = 0.026), which was likely associated with their specific method of practice (acupuncture). Participants in the Others subgroup employed medical assistants significantly more often than the other CM GPs (p = 0.008).

**Table 2:** Structure of the medical practice of general practitioners in the study sample (n = 206).

Characteristics	Total n = 206	TCM/A^1^ n = 73 (35.4%)	HOM^2^ n = 76 (36.9%)	Others^3^ n = 57 (27.7%)	p-value*
Workplace, no. (% per column)					
Single practice	112 (54.4%)	37 (50.7%)	54 (71%)	21 (36.8%)	<0.001^a^
Double/group practice	94 (45.6%)	36 (49.3%)	22 (29%)	36 (63.2%)	
Double/group practice with other CM ^4^ doctors, no. (% per column), n = 94	61 (64.9%)	20 (55.6%)	14 (63.6%)	27 (75.0%)	0.001^a^
Number of doctors in group practice, mean (SD)	3.5 (3.5)	3.8 (3.9)	2.8 (1.5)	3.6 (4)	0.869^b^
Years working at current workplace, mean (SD)	17.8 (11.6)	17 (11.1)	19.3 (11.2)	16.8 (12.6)	0.961^b^
Changed type of practice last 5 years, no. (% per column)	82 (39.8%)	27 (27.4%)	32 (42.1%)	23 (40.4%)	0.778^a^
Single to double/group	17 (20.7%)	7 (26%)	5 (15.6%)	5 (21.7%)	
Double/group to single	34 (41.5%)	8 (29.6%)	20 (62.5%)	6 (26.1%)	
double/group to double/group	19 (23.2%)	8 (29.6%)	2 (6.3%)	9 (39.1%)	
NA	12 (14.6%)	4 (14.8%)	5 (15.6%)	3 (13.1%)	
Change planned for the next years, no. (% per column)	17 (8.3%)	7 (9.6%)	5 (6.6%)	5 (8.8%)	0.803^a^
Single to double/group	10 (58.8%)	3 (42.9%)	3 (60%)	4 (80%)	–
Double/group to single	2 (11.8%)	1 (14.3%)	0 (0%)	1 (20%)	
double/group to double/group	4 (23.5%)	3 (42.9%)	1 (20%)	0 (0%)	
NA	1 (5.9%)	–	1 (20%)	–	
Member of a managed care network, no. (% per column	111 (53.9%)	42 (57.5%)	39 (51.3%)	30 (52.6%)	0.73^a^
Employing medical assistants, no. (% per column)	143 (69.4%)	49 (67.1%)	46 (60.5%)	48 (84.2%)	0.008^a^
NA	2 (1%)	–	1 (1.3%)	1 (1.7%)	
Child and youth care, no. (% per column)	142 (68.9%)	42 (57.5%)	57 (75.0%)	43 (75.4%)	0.026^a^
NA	1 (0.5%)	–	1 (1.3%)	–	
Care for pregnant women, no. (% per column)	32 (15.5%)	13 (17.8%)	8 (10.5%)	11 (19.3%)	0.325^a^
NA	1 (0.5%)	–	1 (1.3%)	–	
Physician drug dispensing, no. (% per column)	63 (30.6%)	24 (32.9%)	21 (27.6%)	18 (31.6%)	0.803^a^
NA	1 (0.5%)	–	1 (1.3%)	–	

*Statistical analysis is conducted using SPSS; NA (Non-Applicable) data points, if present, are always included in calculations.

^a^Chi-Square Test (Pearson Chi-Square, 2-sided).

^b^Linear Regression ANOVA.

^1^Traditional Chinese Medicine/Acupuncture.

^2^Homeopathy.

^3^Anthroposophical Medicine, Phytotherapy, holding > 1 proficiency certificate.

^4^CM Complementary Medicine.

### Workload

At the time of the survey, the average working time was 36 h/week (Table 3). Others GP work significantly more than the other two groups, both in total (p = 0.025) and in administration (p = 0.014). This group also cares more frequently for patients in old people’s home and nursing home (p = 0.006).

**Table 3:** Workload of the general practitioners in the study sample (n = 206).

Characteristics	Total n = 206	TCM/A^1^ n = 73 (35.4%)	HOM^2^ n = 76 (36.9%)	Others^3^ n = 57 (27.7%)	p-value*
Working hours/week now in total, mean (SD)	35.6 (14.6)	34.6 (14.6)	32.7 (13.7)	40.9 (14.6)	0.025^b^
Administration hours/week, mean (SD)	6.9 (5.7)	6.2 (5.5)	6.2 (3.9)	8.8 (7.5)	0.014^b^
Emergency duty days/year, mean (SD)	9.5 (27.6)	8.2 (11.5)	6.4 (12.6)	15.4 (48.6)	0.172^b^
Postgraduate education days/year, mean (SD)	11.4 (8)	10.3 (6.2)	11.7 (8.6)	12.5 (9.1)	0.123^b^
Home visits for patients, no. (% per column)	125 (60.7%)	40 (54.8%)	43 (56.6%)	42 (73.7%)	0.060^a^
Medical care in old people’s home and nursing home, no. (% per column)	101 (49%)	33 (45.2%)	30 (39.5%)	38 (66.7%)	0.006^a^
Availability by phone outside opening hours, no. (% per column)	125 (60.7%)	34 (46.6%)	55 (72.4%)	36 (63.2%)	0.004^a^
NA	1 (0.5%)	–	1 (1.3%)	–	
Home visits outside opening hours, no. (% per column)	102 (49.5%)	28 (38.4%)	43 (56.6%)	31 (54.4%)	0.050^a^
NA	1 (0.5%)	–	1 (1.3%)	–	
Training of medical students, no. (% per column)	55 (26.7%)	23 (31.5%)	13 (17.1%)	19 (33.3%)	0.064^a^
NA	1 (0.5%)	–	1 (1.3%)	–	
Training of residents, no. (% per column)	46 (22.3%)	18 (24.7%)	12 (15.8%)	16 (28.1%)	0.198^a^
NA	1 (0.5%)	1 (1.4%)	–	–	
CON^4^	22 (10.7%)	12 (66.7%)	3 (25%)	7 (43.8%)	
CM^5^	13 (6.3%)	6 (33.3%)	7 (58.3%)	0 (0%)	
Both	11 (5.3%)	0 (0%)	2 (16.7%)	9 (56.2%)	

*Statistical analysis is conducted using SPSS; NA (Non-Applicable) data points, if present, are always included in calculations.

^a^Chi-Square Test (Pearson Chi-Square, 2-sided).

^b^Linear Regression ANOVA.

^1^Traditional Chinese Medicine/Acupuncture.

^2^Homeopathy.

^3^Anthroposophical Medicine, Phytotherapy, holding > 1 proficiency certificate.

^4^CM Complementary Medicine.

^6^CON Conventional Medicine.

Within the subgroups, TCM/A GPs were the least available by phone outside opening hours, whereas HOM GPs showed the highest availability (p = 0.004). Only a minority of the practices provided training for students and/or residents.

### Professional commitment and planning succession

Over the last 5 years, almost half of all CM GPs reduced their working hours, with an average reduction of 13 h/week (Table 4). Two-fifths plan to reduce this in the future, with a mean of 12 h/week. There were no significant differences among the three groups (estimated using linear regression and analysis of variance). The continuous reduction in working hours over time is illustrated in Fig 2. Apart from age, the most frequently cited reasons for reducing working hours were family, illness, and other professional commitments (e.g., new additional employment in a hospital or research activity) (Fig 3). Reducing stress and overwork, improving work-life balance with more time for hobbies and well-being, and avoiding burnout were the most frequently cited “other reasons.”

**Table 4:** Profile of professional commitment and succession planning of the general practitioners in the study sample (n = 206).

Characteristics	Total n = 206	TCM/A^1^ n = 73 (35.4%)	HOM^2^ n = 76 (36.9%)	Others^3^ n = 57 (27.7%)	p-value*
Reduction of working hours in the last 5 years, no. (% per column)	93 (45.1%)	30 (41.1%)	34 (44.7%)	29 (50.9%)	0.539^a^
NA	1 (0.5%)	–	1 (1.3%)	–	
How many hours/week, mean (SD)	12.6 (10.6)	15 (12.2)	10.35 (7.8)	10.7 (10)	0.118^b^
Planning to reduce working hours in the future, no. (% per column)	87 (42.2%)	24 (32.9%)	35 (46.1%)	28 (49.1%)	0.124^a^
How many hours/week, mean (SD)	12.1 (10.8)	10.6 (10)	10.4 (7.8)	15.5 (13.8)	0.096^b^
Giving up working before the statutory retirement age, no. (% per column)	9 (4.4%)	6 (8.2%)	1 (1.3%)	2 (3.5%)	0.033^a^
Work after retirement age, no. (% per column)	38 (18.4%)	8 (11%)	18 (23.7%)	12 (21.1%)	0.004^a^
How many hours/week, mean (SD)	20.1 (8.8)	16 (5.5)	21.8 (9.1)	19.7 (9.6)	0.507^b^
Looked/looking for successor(s), no. (% per column)	54 (26.2%)	14 (19.2%)	21 (27.6%)	19 (33.3%)	0.192^a^
NA	2 (1%)	1 (1.4%)	1 (1.3%)	–	
Found successor(s), n = 54, no. (% per column)	20 (37.0%)	3 (21.4%)	11 (52.4%)	6 (31.6%)	0.156^a^

*Statistical analysis is conducted using SPSS; NA (Non-Applicable) data points, if present, are always included in calculations.

^a^Chi-Square Test (Pearson Chi-Square, 2-sided).

^b^Linear Regression ANOVA.

^1^Traditional Chinese Medicine/Acupuncture.

^2^Homeopathy.

^3^Anthroposophical Medicine, Phytotherapy, holding > 1 proficiency certificate.

Very few CM GPs wanted to retire before the statutory retirement age (4%). However, compared to all CM GPs, approximately double the number of TCM/A GPs desired to retire before that age (p = 0.033). Within the subgroups, approximately 24% of HOM GPs planned to continue working after reaching the retirement age, which is significantly higher than the rest (p = 0.004). Only a quarter of all CM GPs had been looking for successors, and 37% of them were successful. Most were not or only slightly concerned about succession (Fig 4). The most important criterion in the search for a successor was professional qualification. Sex, age, and nationality were found to be irrelevant (Fig 5).

### Optimism for the future

According to the participants’ perception, there was a shortage of all four CM specialties in regional healthcare, particularly GPs, with additional training in homeopathy (Fig 6). Except for HOM GPs (51%), the majority of the other GP subgroups were very optimistic about the future development of their specialization (70–77%) (Fig 7). The future of complementary medicine as a whole was also viewed optimistically by most participants in all three groups (71%).

## Discussion

Our study showed that CM GPs faced the same challenges as the 2015 Swiss Workforce Study GPs, particularly in terms of workforce [2]. Half of them were aged 60 years or older and would retire in the next 5 years. Of all participants in our study, 45% had already reduced their working hours by 13 h/week to 36 h/week at the time of the survey. The main reasons were age, family, illness, and time overload, with a desire for stress reduction and more free time. The fact that 20% of CM GPs stated they wanted to continue working with a planned working time of 20 h/week after retirement would, at best, alleviated and postponed the problem of the shortage of GPs.

There are differences in the care of patients outside practice. CM GPs made fewer home visits and were less likely to care for patients in nursing homes. The lower number of emergency service days may be due to the fact that CM GPs mostly work in urban areas with a higher density of doctors.

Most CM GPs were optimistic about the future (72%), whereas participants in the 2015 Swiss Workforce Study were pessimistic (62%). A larger number of GPs in the 2015 Swiss Workforce Study wanted to end their practice before the retirement age or were planning to reduce working hours than those in our survey (22% vs. 5%, 51% vs. 42%). Given the age structure of both study populations, the reduction in working hours owing to age or health problems is understandable. Nevertheless, these doctors continue to work and make valuable contributions to primary care within the scope of their capabilities. Other reasons for reducing working hours, such as reducing stress or preventing exhaustion, reflect the increasingly stressful working conditions of Swiss GPs.

These differences may also be related to the slightly higher job satisfaction of CM GPs [17]. Additionally, owing to their additional qualifications in CM therapy, CM GPs can reduce their conventional GP workload and mainly treat patients using CM. This contradicts the fact that CM GPs who work after the statutory retirement age alleviate the shortage of Swiss GPs. However, specific data regarding this issue are lacking. In our view, this would not be detrimental to conventional medicine GPs but rather benefit them by allowing them to treat different patient groups, such as patients with chronic pain and uncomplicated infections.

A comparison with information from the 2002 Evaluation of Complementary Medicine program showed an increase in the number of female doctors (especially TCM/A GPs) and an increase in group practices, in line with the general trend in Swiss primary care [6,18]. Conversely, 40% of CM GPs have changed their practice type in the last 5 years, mostly from a double or group practice to a single practice. HOM GPs are increasingly working in single practices. We assume that HOM GPs have often been in small-group practice with a colleague, and after the colleague leaves, they continue to work alone because of the lack of a successor. The increase in the average age, particularly among HOM GPs from 52 to 63 years, is a concern and an indication of the shortage of younger doctors.

### Limitations and strengths

This study has limitations, including its cross-sectional design and reliance on self-reported data. However, the proposed design was the best choice in the absence of a register. For data protection reasons, we were unable to obtain the addresses of all CM doctors from the Swiss Medical Association. Therefore, the survey was sent only to the members of the four professional associations [9–12]. With a response rate of 27% and an inclusion rate of 19% in the analysis, the study results are probably not fully representative of all professional groups. However, reviews indicate that such response rates do not lead to selection bias in surveys with physicians [19,20]. The answers on future working hours and the development of specialization only provide indications but may differ from actual development. The 2015 Swiss Workforce Study did not exclude GPs with additional training in CM; therefore, CM GPs could be represented in both study populations, once as GPs only and once as CM GPs. This led to limitations when comparing the two studies.

Our survey collected comprehensive information on the professional group of CM GPs and can, therefore, serve as a basis for further analysis. Future research on these fundamentals could explore longitudinal trends in workforce dynamics and succession planning among CM GPs, as well as professional satisfaction within this cohort.

### Implications

The findings of this study have implications for workforce planning and policy development in Swiss primary care centers. Addressing the shortage of GPs requires targeted strategies to attract and retain practitioners, including support for succession planning initiatives and investment in training and education. A stronger commitment to training residents in CM GP practices combined with working models that consider feminization and the desire for a good work-life balance is urgently needed [21]. To avoid jeopardizing primary medical care, strengthening partnerships between CM practitioners and conventional medical professionals is essential, as many similarities exist between the two workforces.

Traditionally, there is a high demand for CM in Switzerland [22]. With the shortage of CM GPs that already exists and will become even more acute in the future, there is a risk of patients switching to nonphysician therapists (Alternative Medicine Therapists). This can lead to less qualified treatment and financial burden. Treatments by Alternative Medicine Therapists are not reimbursed by mandatory basic insurance.

## Conclusion

This study provides valuable insights into the workforce, succession planning, and the outlook of Swiss GPs with additional training in CM. As primary care providers in Switzerland, CM GPs face the same challenges as their colleagues in conventional medicine. The trend towards reduced working hours for CM GPs, coupled with an aging workforce, emphasizes the importance of implementing national measures to enhance GP training and improve working conditions—requiring collaboration from all GPs involved in basic medical care in Switzerland.

## Supporting information

S1 Questionnaire English translation of the German-language questionnaire. (DOCX)
