# Supplementary material for: Workforce, succession planning, and optimism for the future among Swiss general practitioners with additional training in complementary medicine in 2016: A nationwide cross-sectional study
Source: PLoS One. 2026 Feb 6;21(2):e0342226. doi: 10.1371/journal.pone.0342226 (PMC12880634; doi:10.1371/journal.pone.0342226)
Supplement: S1 Questionnaire — (DOCX) [file pone.0342226.s001.docx]

**S 1. English translation of the German-language** **questionnaire**

Dear participants

Thank you for your interest in the "Workforce Study of Complementary Medicine". The questionnaire takes about 20 minutes.

The participation is anonymous, and your details are treated confidentially.

Questions about your person

Nr. 1 Sex


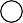
 male


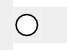


female

Nr. 2 Year of birth

Nr. 3 Nationality


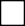
 Switzerland

France


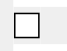

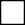


Germany

Austria


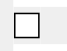

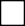


Italy


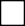
 Liechtenstein


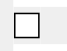


Other:

Nr. 4 In which year did you complete the state examination?

Questions about your occupational activity

Nr. 5 Which FMH-certified specialty do you hold?

Multiple selection possible.


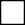
 Medical Practitioner
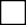
 Cardiology
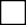
 Pathology

Allergology and Clinical Immunology

Pediatrics

Pharmaceutical Medicine

General Internal Medicine Child and Adolescent

Psychiatry and Psychotherapy

Physical Medicine and Rehabilitation

Anaesthesiology

Pediatric Surgery

Plastic, Reconstructive und Aesthetic Surgery

Angiology Clinical Pharmacology and Toxicology

Pneumology

Occupational medicine

Medical Genetics

Prevention and Public Health

Surgery Medical Oncology Psychiatry and Psychotherapy

Dermatology and Venerology

Cranio Maxillofacial Surgery

Radiology

Endocrinology / Diabetology Nephrology Radio-Oncology / Radiation Therapy


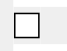

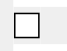

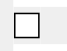


Gastroenterology

Forensic Medicine

Neurosurgery


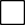
 Gynaecologie and Obstetrics
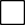
 Neurology
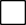
 Rheumatology


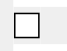

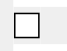

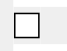


Hematology

Tropical und Travel Medicine

Neuropathology


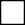
 Hand Surgery
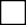
 Nuclear Medicine
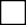
 Urology

Cardiac and Thoracic Vascular Surgery

Ophthalmology


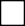
 Infectiology
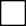
 ORL

Intensive Care

Orthopedic Surgery and Traumatology of the Musculoskeletal System

Nr. 6 Regarding your FMH proficiency certificates:

In which year did you complete the additional training and when did you obtain the proficiency certificates?

Enter the year(s) according to your proficiency certificate(s).

Anthroposophic Medicine

Homeopathy Neural Therapy Phytotherapy TCM/Acupuncture

Year additional training completed:

Year proficiency certificate acquired:

Nr. 7 Have you completed further training in complementary medicine without obtaining a proficiency certificate?


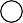
 Yes
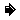
Please continue with question 8.


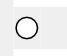

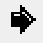


Please continue with question 9.

No

Nr. 8 What training in complementary medicine have you completed without obtaining a proficiency certificate?

Multiple selection possible.


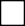
 Anthroposophic Medicine

Neural Therapie


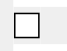

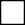


Homeopathy

TCM/Acupuncture


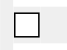

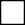


Phytotherapy

Nr. 9 What kinds of work do you currently carry out in your practice?

Single selection.


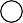
 Mainly complementary medicine

Certified specialty and complementary medicine


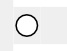

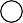


General practice and complementary medicine


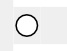


Mainly conventional medicine

Questions on practice structure

Nr. 10 Do you work in a:

Single selection.


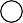
 Practice


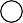
 Hospital/Clinic Please continue with question 66.


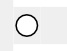

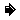


Practice and hospital/clinic

Nr. 11 What type of practice do you work in?

Single selection.


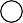
 in a single practice


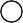


in a group practice with physicians

Nr. 12 Are you:

Single selection.


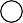
 self-employed


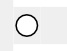


employed

Nr. 13 Since when have you been working in this practice?

Enter year.

Nr.: 14 Have you been practicing in another type of practice before this date?


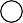
 Yes
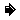
Please continue with question 15.


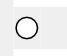

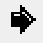


Please continue with question 16.

No

Nr. 15 Change from:

Previous type of practice.


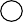
 Single practice


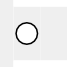


Group practice with physicians

to:

Current type of practice.


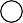
 Single practice


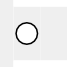


Group practice with physicians

Nr. 16 Are you planning to change your type of practice in the future?


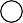
 Yes
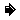
Please continue with question 17.


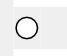

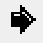


Please continue with question 19.

No

Nr.: 17 In which year are you planning a change?

Enter year, if necessary.


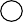


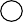
 Time uncertain

Nr. 18 Change from:

Current type of practice.


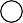
 Single practice


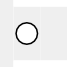


Group practice with physicians

to:

Future type of practice.


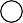
 Single practice


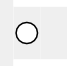


Group practice with physicians

Nr. 19 Where do you practice?

Single selection.


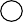
 in a rural region (<5‘000 inhabitants)


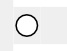


in a small town (5'000 - 10'000 inhabitants)


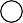
 in a town (> 10'000 inhabitants)

Nr. 20 Do you employ medical practice assistants?

Enter job percentage as whole number, if necessary.

Yes, percent by position.


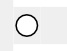

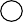


No

Nr. 21 Which of the following services do you offer personally in your practice?

Multiple selection possible.

Care / Examination of children and adolescents

Gynecological Examination

Spirometry Full pharmaceutical dispensation (physicians with dispensary)

Sonography Partial pharmaceutical dispensation

(e.g. first submission)

Prenatal care
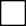
 24h blood pressure
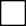
 Only emergency medication measurement


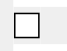

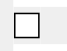


Radiography

Point of Care Laboratory

ECG
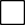
 Wound care / Suture


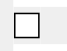

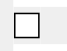


Casts

Ergometry

Treatment of fractures

Nr. 22 Does your practice offer physiotherapy?


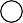
 Yes


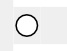
No

Nr. 23 Are other doctors in your practice with a proficiency certificate in complementary medicine?


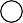
 Yes
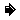
Please continue with question 24.


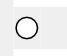

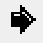


Please continue with question 26.

No

Nr. 24 How many physicians hold a proficiency certificate in complementary medicine in your practice?

Enter count as whole number.

physicians

Nr. 25 ... and which specialisation?

Multiple selection possible.


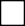
 Anthroposophic Medicine

Neural Therapy


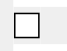

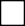


Homeopathy

TCM/Acupuncture


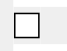

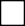


Phytotherapy

Nr. 26 Do you keep an electronic patient record?


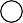
 Yes
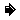
Please continue with question 27.


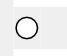

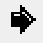


Please continue with question 28.

No

Nr. 27 In which year did you switch from paper patient records to electronic patient records?

Enter year.

Nr. 28 Are you planning to switch to electronic patient records?


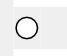

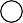
 Yes
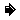
Please continue with question 29.


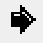
No Please continue with question 30.

Nr. 29 In which year are you planning to switch from paper patient records to electronic patient records?

Enter year, if necessary.


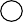


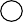
 Time uncertain

Nr. 30 Can you imagine a changeover to the electronic patient record?


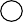
 Yes


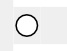


No

Nr. 31 Are you a member of a physician’s network?

Yes

No

Questions about work

*Working hours:*

Nr. 32 How many hours do you work on average in your practice per week?

Enter hours as whole numbers.

hours/week

Nr. 33 How many hours of it do you need for administrative work per week?

Enter hours as whole number.

hours/week

*Home visits:*

Nr. 34 Do you make home visits during working hours?

Yes

No

Nr. 35 Do you treat patients in retirement and nursing homes?

Yes

No

*Emergency services:*

Nr. 36 How many days of emergency and on-call services do you perform on average in the regional medical emergency service per year?

Enter days as whole number.

days/year

Nr. 37 Are you normally available for phone consultations for your patients outside your working hours?

Yes

No

Nr. 38 Are you normally available for services (practice, home visit, retirement and nursing home, etc.) for your patients outside your working hours?

Yes

No

*Absence of practice:*

Nr. 39 How many working days were you absent due to training in 2016?

Enter days as whole number.

days

Nr. 40 How many working days were you absent due to holidays in 2016?

Enter days as whole number.

days

*Additional professional activities:*

Nr. 41 Do you teach medical students in your practice?

(e.g. GP module of the Bern Institute of Family Medicine BIHAM)

Yes

No

Nr. 42 Do you offer practice assistant positions for:

Single selection.

candidates for certified specialties

both

candidates for proficiency certificates

none

Nr. 43 Do you participate in quality circles?

Single selection.

Yes, I completed the moderator training.

Yes (without moderator training).

No.

Nr. 44 Do you participate in balint groups?

Yes

No

Nr. 45 Do you participate in supervision and/or intervision groups?

Yes Please continue with question 46.

Please continue with question 47.

No

Nr. 46 In which supervision and/or intervision groups do you participate?

Multiple selection possible.

Anthroposophic Medicine

Neural Therapy

Homeopathy

TCM/Acupuncture

Phytotherapy

Other

Nr. 47 Are you active in professional policies as a board member or in a comission?

Single selection.

Yes, in conventional medicine.

Yes, in complementary medicine.

Yes, both.

No

Nr. 48 Do you perform any official duties (e.g. school physician function, etc.)?

Yes

No

Nr. 49 Are there any other secondary activities that have not been mentioned here that you perform?

Free text entry possible.

Yes, namely

No

Nr. 50 How much is your regular KVG-independent income per year? (e.g. medical officer, school physician, network, emergency service compensation, VVG, etc.)

Single selection.

< 8'000 CHF

> 8'000 CHF

Questions about the work structure

Nr. 51 Have you reduced your working hours in your practice in the last 5 years?

Yes Please continue with question 52.

No Please continue with question 54.

Nr. 52 How many working hours per week have you reduced?

Enter hours as whole number.

hours/week

Nr. 53 What were the reasons for the reduction?

Multiple selection possible.

Family

Illness

Age

(Professional) Policies

Secondary professional activity

Other:

Nr. 54 Are you planning to reduce your working hours in the future?

Yes Please continue with question 55.

Please continue with question 61.

No

Nr. 55 In which year?

Enter year, if necessary.

Time uncertain

Nr. 56 How many working hours per week are you planning to reduce?

Enter hours as whole number.

hours/week

Nr. 57 Are you planning to give up your work as a practitioner before the statutory retirement age?

Enter age as whole number, if necessary.

Yes, at the age of .

No.

Nr. 58 Are you planning to continue working in your practice even after you have reached the statutory retirement age?

Yes Please continue with question 59.

Please continue with question 61.

No

Nr. 59 How many working hours per week?

Enter hours as whole numbers.

hours/week

Nr. 60 How many hours of this are for … ?

Complementary medicine: hours/week

Conventional medicine: hours/week

Nr. 61 Do you worry about the succession or handover of your practice?

not at all

rather much

a little

very much

Nr. 62 Have you been looking for or are you looking for successors for your practice?

Enter months as whole number, if necessary.

Yes, I am looking for month now. Please continue with question 63.

Yes, I have looked for months. Please continue with question 63.

Please continue with question 66.

No

Nr. 63 Have you found a successor?

Enter count of successors as whole number, if necessary.

Yes, I have found successors. (e.g. part-time employment)

No.

Nr. 64 What was or is especially important to you in this search?

Mark a gradation for each criterion.

| Education in complementary medicine (without proficiency certificate) | not important | less important | quite important | important |
| --- | --- | --- | --- | --- |
| Proficiency certificate in complementary medicine | not important | less important | quite important | important |
| Certified specialty | not important | less important | quite important | important |
| Gender | not important | less important | quite important | important |
| Year of birth | not important | less important | quite important | important |
| Nationality | not important | less important | quite important | important |

Nr. 65 Other criteria that were or are important to you in your search:

If not mentioned above, enter here..

Questions about health policy

Nr. 66 Is there a shortage of physicians in complementary medicine in your region?

Yes Please continue with question 67.

Please continue with question 69.

No

I don’t know Please continue with question 69.

Nr. 67 Wherein lies a shortage?

Multiple selection possible.

Anthroposophic Medicine

Neural Therapy

Homeopathy

TCM/Acupuncture

Phytotherapy

Nr. 68 How does this shortage manifest itself?

Enter free text.

Nr. 69 Are you optimistic about the future of YOUR specific proficiency certificate?

not at all

little

rather

very

Nr. 70 Are you optimistic about the future of complementary medicine as a whole?

not at all

little

rather

very

Nr. 71 In May 2009, the constitutional article on complementary medicine was adopted.

How satisfied are you with the implementation so far?

unsatisfied

rather unsatisfied

rather satisfied

very satisfied

Nr. 72 Have you already participated in complementary medicine research projects?

Yes

No

Nr. 73 Is there an interest in participating in complementary medicine research?

Yes

No

Nr. 74 Would you like to be registered in a research pool with physicians of complementary medicine to strengthen future research projects in complementary medicine?

Yes Please continue with question 75.

Please continue with question 76.

No

Nr. 75

Registration research pool:

Therefor you need the following password: **ikom_2017**

<http://www.ikom.unibe.ch/forschung/index_ger.html>

Nr. 76 Comments and feedback on the questionnaire:

Enter free text.

**Thank you for your valuable participation!**

You have successfully completed the questionnaire.

You will be informed about the results after publication with a newsletter of your professional society.

With the following link, you can download the corresponding form for educational confirmation: For this you need the following password: **ikom_2017**

[**http://www.ikom.unibe.ch/forschung/index_ger.html**](http://www.ikom.unibe.ch/forschung/index_ger.html)
